# The daily minimum leaf turgor pressure can represent the water status of apple trees under drip irrigation

**DOI:** 10.3389/fpls.2024.1516824

**Published:** 2024-12-18

**Authors:** Ruixia Chen, Quanyue Xu, Junliang Wan, Nan Jiang, Juanjuan Ma, Lijian Zheng

**Affiliations:** ^1^ College of Water Resources Science and Engineering, Taiyuan University of Technology, Taiyuan, China; ^2^ Shanxi Key Laboratory of Cooperative Utilization for Basin Water Resources, Taiyuan, China

**Keywords:** apple tree, leaf turgor pressure, stem flow rate, signal intensity, water status

## Abstract

**Introduction:**

Accurate diagnosis of the water status of fruit trees is a prerequisite for precise irrigation. Measurement of leaf turgor pressure provides a means to explore the water utilization mechanisms of fruit trees and their responses to water stress. However, there are few studies on the use of daily minimum leaf turgor pressure (Ppmax) to indicate water information in apple tree.

**Methods:**

We monitored Ppmax, stem flow rate (Vstem), leaf water potential, soil water, and the main meteorological factors under two drip irrigation levels (fully irrigated and under-irrigated) to elucidate the temporal dynamics of the daily leaf turgor minimum of apple trees and diagnose the water status of fruit trees.

**Results:**

The results showed that soil water deficiency could reduce leaf turgor pressure and increase Ppmax. In both years, the signal intensity and sensitivity of Ppmax were better than those of Vstem, and the sensitivity was 3.31 and 2.94 times of Vstem, respectively. Compared to Ppmax, Vstem had a higher correlation with meteorological factors and was more affected by meteorological conditions, independent of irrigation treatment. Ppmax was significantly negatively correlated with soil and leaf water potential, and its correlation coefficient was 2.58–4.89 times higher than that between Vstem and both.

**Discussion:**

These results not only enhance our understanding of the water regulation strategies employed by apple trees under drought conditions but also provide a theoretical foundation for developing efficient water-saving practices and precision irrigation strategies for fruit trees.

## 1 Introduction

Apples are mainly produced in arid and semi-arid regions. Apple production in the Loess Plateau region has rapidly increased in recent years, accounting for one-quarter of the total apple production in China (Huang et al., 2021). However, the region’s lack of water resources and uneven rainfall distribution over time and space have greatly limited apple production. Moreover, unreasonable irrigation methods are usually adopted to achieve a high yield in orchards, leading to wastage of water resources and reduces the water-use efficiency of fruit trees (Wen et al., 2024). Therefore, developing water-saving irrigation technology is fundamental to promote the precise irrigation of orchards and efficient water use by fruit trees (Arbizu-Milagro et al., 2022). Among them, accurate fruit tree water information acquisition is the key to formulating precision irrigation strategies (Faghih et al., 2021).

Many studies have applied environmental parameters (such as soil moisture content and atmospheric temperature) and water physiological indicators (such as trunk diameter, stem and leaf water potential, and leaf thickness) to the water diagnosis of fruit trees (Afzal et al., 2017; Mota et al., 2018; Li et al., 2020; Tsouliasu et al., 2020). However, problems, such as complex processes and unstable accuracy, still exist in the realization of nondestructive continuous monitoring. Stems and leaves are the main organs of fruit tree growth and water transport, and are usually used to indicate the water status of fruit trees. Previous studies have shown that stem flow can provide information about the water status of plants because high transpiration rates lead to more negative tension within the stem xylem (De Swaef and Steppe, 2010), and it is able to directly feedback the effects of plant water on the environment (Wheeler et al., 2023; Zhao et al., 2023). Some researchers have used the stem flow index to evaluate the water-use strategy of apple trees, and the results have shown a correlation between stem flow and the response of fruit trees to drought. Moreover, stem flow technology can be used to quantify the sensitivity and resilience of apple rootstocks to summer water deficits (Muchena et al., 2020; Mobe et al., 2020; Ntshidi et al., 2021). However, other studies found that stem flow is significantly affected by meteorological factors, which may cause errors in the judgment of water information, resulting in insufficient irrigation (Corell et al., 2013). Therefore, whether stem flow accurately represents fruit tree water remains controversial. On this basis, finding a water physiological index with a simple operation and high accuracy is necessary.

At the plant scale, leaf turgor pressure plays a decisive role in stomatal opening and closing and the water-carbon cycle (Knipfer et al., 2020). The relative leaf turgor value (Pp), monitored using a noninvasive magnetic patch-clamp pressure probe (LPCP), can accurately characterize leaf water status (Zimmermann et al., 2008). Many scholars have used this technology to study woody plants, such as olive trees (Marino et al., 2016), grape trees (Rüger et al., 2010a), banana trees (Zimmermann et al., 2010) and persimmon trees (Martinez-Gimeno et al., 2017), as well as crops, such as wheat (Bramley et al., 2013) and pepper (Camoglu et al., 2021), indicating that the leaf turgor pressure change pattern recorded by the LPCP can reflect changes in plant water status. In addition, LPCP technology can help determine the effect of water stress on leaf turgor pressure, as shown by Marino et al. (2021). It was also more sensitive in diagnosing water deficit than other physiological indicators, such as relative water content of the leaves and slight changes in the trunk diameter. These results indicate that leaf turgor pressure has the potential to guide precise irrigation of trees (Fernandes et al., 2017). Scholars have proposed using relative leaf turgor pressure-derived parameters, including daily minimum turgor pressure (Ppmax), daily maximum turgor pressure (Ppmin), and turgor pressure recovery time to quantify changes in leaf turgor pressure more accurately (Rüger et al., 2010b; Riboldi et al., 2016). Xu et al. (2024) analyzed the response of relative leaf turgor pressure parameters at different positions of the apple tree canopy to changes in soil moisture and determined Ppmax as the optimal parameter. However, further investigation is required to determine whether Ppmax can accurately characterize plant water physiology.

In addition, fruit tree growth and the external environment affect the real-time output data of leaf turgor pressure (Gokhan et al., 2021). It is necessary to eliminate the deviation caused by external factors to clarify the accuracy of Ppmax characterization of the apple tree water status and achieve accurate irrigation of apple trees. The signal strength theory is a method for evaluating the sensitivity of plant water diagnostic indicators, which can eliminate error sources and reduce the influence of meteorological factors on the data (Du et al., 2017; Ru et al., 2021). This method has been applied to analyze the response of indicators such as stem flow rate and daily shrinkage of the trunk diameter of apples, peaches, and olive trees to soil water change (Conejero et al., 2007; Fernandez et al., 2011). However, no studies have used this method to evaluate the sensitivity of Pp or its parameters.

Therefore, a two-year field experiment was conducted in apple orchards in the Loess Plateau area with dwarf anvil apple trees treated with different drip irrigation levels. The objectives of the study were to (1) investigate the temporal dynamics of the daily minimum leaf turgor pressure under different soil water conditions and (2) clarify the accuracy of Ppmax in indicating the water status of apple trees under drip irrigation.

## 2 Materials and methods

### 2.1 Experiment site

The experiment was conducted at the Fruit Research Institute of Shanxi Agricultural University (112° 32’ E, 37° 23’ N) in 2022 and 2023. The altitude of the experimental site is 781.9 m, the average annual temperature is 9.8 °C, and the frost-free period is 175 days a year. The spring is dry and rainless, the summer and autumn rainfall is high and concentrated, and the annual average rainfall is 459.6 mm, indicating a temperate continental climate. The 0–200 cm soil layer in this area is silty loam, with an average bulk density of 1.47g‧cm^-3^, an average field water holding ratio (θ_f_) of 30%, and a saturated water content of 49.8%. The fruit trees were covered with polypropylene black ground cloth to reduce ground evaporation and weed growth.

### 2.2 Irrigation treatment

Two drip irrigation levels were set up in this experiment, fully irrigated treatment (WW), in which the upper and lower limits of soil water content were 70%θ_f_–100%θ_f_, respectively, and under-irrigated treatment (WS), in which the upper and lower limits of soil water content were 50%θ_f_–70%θ_f_, respectively. A single fruit tree was used as the experimental plot, and three replicates were used for each treatment. During the experiment, the soil moisture content was measured weekly, and when the soil moisture reached the lower limit, irrigation was carried out, and water was injected into the upper limit of soil moisture. Orchard management was consistent with the local areas. According to the growth conditions of local apple trees, it is divided into three growth periods: the growth period of new shoots (May 28–July 4), the fruit expansion period (July 5–September 20), and the fruit maturity period (September 21–October 6) in 2022. The growth period of new shoots (May 28–July 10), the fruit expansion period (July 11–September 24), and the fruit maturity period (September 25–October 10) in 2023.

### 2.3 Measurement

#### 2.3.1 Meteorological and soil moisture

Experiment area adopts Adcon-Ws wireless automatic weather station (ADCON, Germany) to monitor meteorological factors, including rainfall (P, mm), solar radiation (Rs, W‧m^-2^), atmospheric temperature (Ta, °C) and relative humidity (RH, %). The output step of all monitored data is set to 15 minutes. The saturated vapor pressure difference (VPD, kPa) was calculated using the method described by Buck (1981).

Soil moisture content was monitored 60cm away from the trunk on the east side of each tree. The soil moisture content in the 0–100 cm soil layer was monitored using time-domain reflectometry (IMKO Micromodultechnik GmbH, Ettlingen, Germany). The determination interval was 7d and additional measurements were taken before and after rainfall and irrigation.

The soil water potential of the eastern side of the fruit tree 40cm away from the trunk was measured by WP4C water potential instrument (METER Group Inc, Pullman, USA). The soil depth was 0-100cm, and every 20cm was a soil layer. The measured interval was consistent with the soil moisture content.

#### 2.3.2 Leaf turgor pressure

The leaf turgor pressure probe (LPCP, YARA-ZIM Plant Technology GmbH, Hennigsdorf, Germany) was used to measure the leaf turgor pressure of the apple trees. Three trees were selected per treatment. Before the start of the experiment, leaves with good growth in the eastern, radial and vertical middle of the canopy were selected, and leaf turgor pressure probes were installed on the leaves according to the method of Bramley et al. (2013), avoiding leaf veins. The probe was connected to the CR1000X data acquisition system and set to record the data every 5 min. The relationship between the probe output relative leaf turgor pressure (Pp) and real leaf turgor pressure (Pc) is consistent with Equation 1 (Zimmermann et al., 2008).


(1) $$Pp={\left(\frac{b}{aPc+b}\right)}^{1/a}\cdot Fa\cdot Pclamp$$

Where, a and b are the constants of a single leaf property. Pclamp is the initial magnetic pressure applied by the probe to the blade. Fa is the pressure attenuation coefficient.

In this study, daily maximum Pp (Ppmax) was selected for analysis. The calculation methods of signal strength and sensitivity of Ppmax were as follows.


(2) $$SI_{Ppmax}=\frac{Ppmax_{WW}}{Ppmax_{WS}}$$

(3) $$\;S_{Ppmax}=\frac{SI_{Ppmax}}{CV_{Ppmax}}$$

Where, SI_Ppmax_ is the signal strength of Ppmax. Ppmax_WW_ and Ppmax_WS_ are Ppmax under fully irrigated and under-irrigated treatment, respectively. S_Ppmax_ is the sensitivity of Ppmax and CV_Ppmax_ is the coefficient of variation of Ppmax.

#### 2.3.3 Stem flow

The stem flow of fruit trees installed with a leaf turgor probe was monitored using plant thermal diffusion stem flow meter (TDP, Shiyutong GmbH, Beijing, China), and the data were recorded every 30min. The daily sap flow rate (Vstem) of apple trees was calculated based on the collected data. The signal intensity of the stem flow rate (SI_Vstem_) was the ratio of Vstem in the under-irrigated treatment to Vstem in the fully irrigated treatment. Sensitivity was calculated in the same manner as Ppmax sensitivity.

#### 2.3.4 Leaf water potential

Leaf water potential (Ψ_leaf_) of apple tree with good growth was measured by WP4C water potential instrument (METER Group Inc, Pullman, USA) at 6:00, including three repetitions, with a frequency of 7d.

### 2.4 Data analysis

Origin 2021 (OriginLab, USA) was used for the data processing and mapping. One-way ANOVA was used to analyze the significance of the SI_Ppmax_ and SI_Vstem_ (n=3). The correlation between Ppmax, Vstem, meteorological factors, soil moisture, and leaf water potential tested using linear regression. In the analysis of the relationship between Ppmax, Vstem and meteorological factors, the data of Ppmax, Vstem, solar radiation (Rs), atmospheric temperature (Ta), relative humidity (RH), and saturated water vapor pressure difference (VPD) on sunny days were selected, and the data of Ppmax, Vstem, and rainfall (P) on rainy days were selected over two years.

In this study, the frequency analysis of rainfall data in the recent 20 years in the test site was carried out, and the frequency curve with the horizontal axis as frequency and the vertical axis as total rainfall (P) was drawn. Each hydrological year type can be obtained by the curve, namely, the wet year (P < 530.5mm), the normal year (530.5mm< P <367.0mm) and the dry year (P > 367.0mm).

## 3 Results

### 3.1 Changes in meteorological conditions

**Figure 1:**
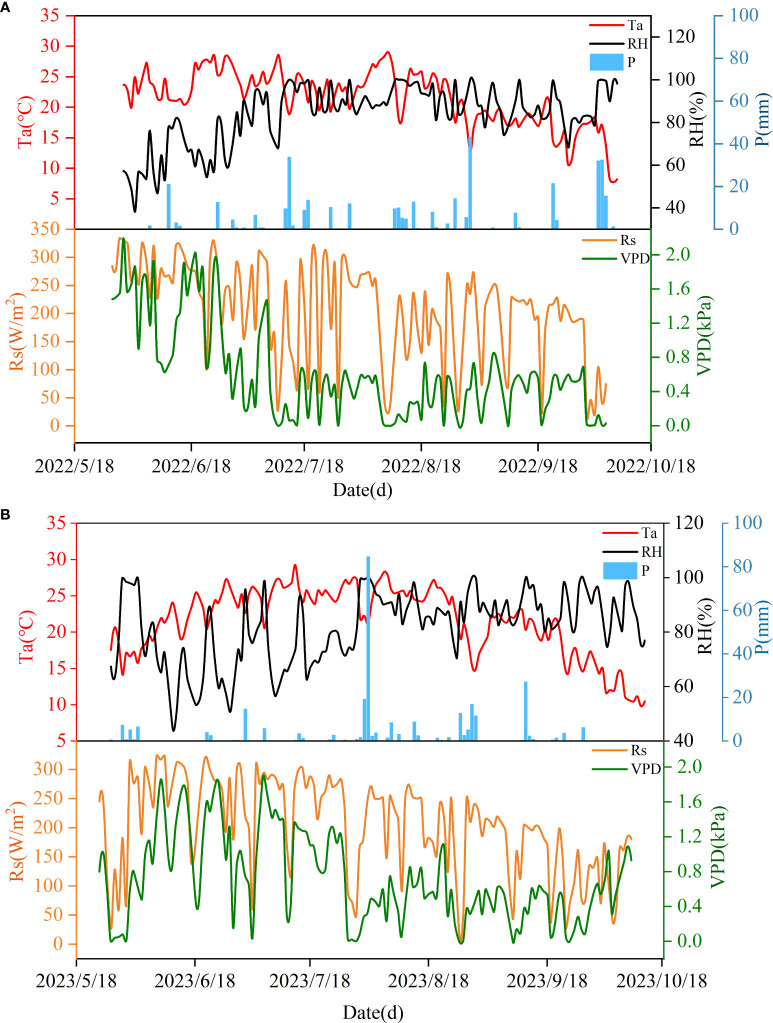
Changes of temperature (Ta), relative humidity (RH), rainfall (P), solar radiation (Rs) and saturated water vapor pressure difference (VPD) during the two-year experiment. **(A)** Meteorological conditions during the 2022 trial period. **(B)** Meteorological conditions during the 2023 trial period.

During the two experimental years, the change patterns of meteorological conditions were similar (**Figure 1**). Specifically, Ta showed a trend of first increasing and then decreasing as apple tree growth progressed. The average temperature during the whole growth period of apple trees was 21.05 °C and 21.53 °C in 2022 and 2023, respectively. The highest temperature was reached in early August (29.01 °C) and mid-July (29.27 °C). The lowest temperatures in both years were recorded at the end of the reproductive period. During the two-year growth period, the RH showed a pattern of increasing fluctuation in the early period and stable fluctuation in the later period, and the relative humidity had a larger range of change in the early period of 2023. The variation laws of Rs and VPD were opposite; both gradually decreased with increasing time, and the fluctuation range was large. The years 2022 and 2023 are normal and dry years, respectively. The cumulative rainfall during the whole growth period of apple trees was 378.00 mm and 295.62 mm.

### 3.2 Change of Ppmax growth period of apple tree

**Figure 2:**
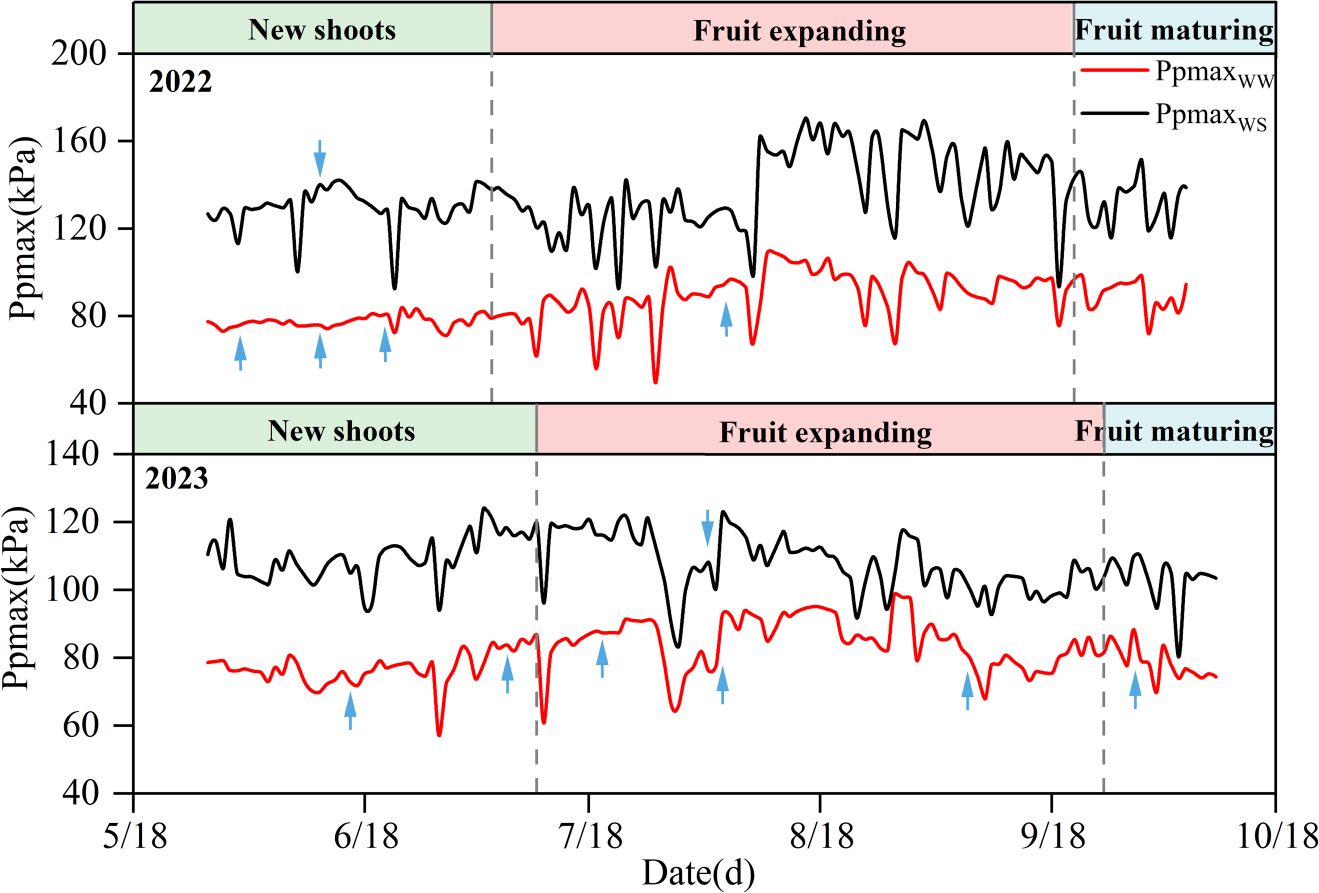
Changes of Ppmax in 2022 and 2023. The blue arrow shows the irrigation time.

As shown in **Figure 2**, there were differences in the Ppmax between the different irrigation treatments during the two experimental years. The Ppmax of the WS treatment was higher than that of the WW treatment, and the average Ppmax of the WS treatment was 1.62 and 1.27 times that of the WW treatment in 2022 and 2023, respectively. During the experiment, with a decrease in soil water content, the leaf turgor pressure of apple trees decreased, and Ppmax increased. When irrigation or rainfall occurred, the leaf water of the fruit tree was supplemented, and Ppmax decreased sharply and then returned to normal levels. In the two years, Ppmax was reduced by a maximum of 10.58% and 10.45% in the WW treatment after irrigation, compared to that before irrigation. However, Ppmax of the WS treatment decreased by 1.65% and 7.39%, respectively. The effect of rainfall on the Ppmax of apple trees was higher than that of irrigation, which was reflected by the maximum reduction in Ppmax of the WW and WS treatments by 22.03% and 18.02%, respectively, over the two years.

In 2022 and 2023, with the advancement of the growth period, the Ppmax of apple trees showed a trend of first increasing and then decreasing (**Table 1**), with stable fluctuations in the early stage and sharp fluctuations in the late stage (**Figure 2**). In the new shoots growth stage in 2022, the soil moisture under WW treatment was sufficient, the leaf turgor pressure of fruit trees remained stable, and Ppmax fluctuated in the range of 71.09–83.77 kPa, while Ppmax under WS treatment increased slightly (except for rainfall and irrigation periods). The Ppmax of the two treatments entered the severe fluctuation stage at the fruit expansion stage and increased significantly, reaching the highest value in mid-August (fruit expansion stage), increasing by 41.45% and 34.31%, respectively, compared with the initial observation value. At the fruit maturity stage, Ppmax in the WW treatment gradually stabilized, whereas that in the WS treatment was still fluctuating and showed a small decrease. By 2023, the changes in Ppmax were similar between the WW and WS treatments. At the early stage of the experiment, the soil moisture was sufficient, and the Ppmax of the two treatments remained in a stable fluctuation range. With the growth of fruit trees, Ppmax increases during the late stage of flourishing of the new shoots. Upon entering the fruit expansion stage, the fluctuation range of Ppmax increased for both treatments. On July 30, rainfall was 84.68mm, resulting in the lowest Ppmax values of the WW and WS treatments, which were 65.64 and 80.75 kPa, and decreased by 17.79% and 14.04%, respectively, compared with the Ppmax before the rain. The Ppmax of the two treatments reached a maximum value at the end of August, which was 1.26 and 1.32 times of the initial value, respectively. At the end of the growth period, Ppmax in both treatments decreased and stabilized at maturity.

**Table 1:** Daily average Ppmax of each growth period in 2022 and 2023.

		Ppmax_WW_ (kPa)	Ppmax_WS_ (kPa)
2022	New shoots	77.22	129.25
	Fruit expanding	89.68	137.97
	Fruit maturing	79.76	132.30
	Whole growth season	82.22	133.17
2023	New shoots	77.86	99.60
	Fruit expanding	85.60	105.71
	Fruit maturing	79.00	103.28
	Whole growth season	80.82	102.86

### 3.3 Difference in signal strength between Ppmax and Vstem

The mean daily signal intensities of Ppmax (SI_Ppmax_) and Vstem (SI_Vstem_) differed significantly between 2022 and 2023 (**Table 2**). In 2022, SI_Ppmax_ gradually increased during the growing season of apple trees. The Ppmax signal value of each stage was 3.25–4.02 times that of SI_Vstem_, and the difference between the two reached a very significant level at the fruit maturity stage and the entire growth stage (*p* < 0.01). The variation range of SI_Vstem_ during the entire growth period of the apple trees was 0.15, which was much lower than that of SI_Ppmax_ (0.43). In addition, under the influence of frequent rainfall, the variability of Ppmax in the WS treatment during the reproductive growth period increased to varying degrees compared with that in the nutrient stage. The CV value of Ppmax in the fruit maturity period was the highest, increasing by 61.29% compared with that in the new shoot growth stage. Ppmax sensitivity tended to decrease during the later stages of growth. However, although the lower variability of Vstem during fruit maturity resulted in a lower CV than that of Ppmax for the entire growth period, it also resulted in a 3.31-fold reduction in sensitivity owing to its smaller signal value. In 2023, SI_Ppmax_ first increased and then decreased during the entire apple tree growth period. The mean SI_Ppmax_ of the entire growth period was significantly higher than that of SI_Vstem_ and increased by 62.62% compared with SI_Vstem_. In contrast to 2022, the CV value of Ppmax in 2023 was lower than that of Vstem, and its sensitivity was 2.94 times higher than that of Vstem.

**Table 2:** Ppmax and Vstem signal strength, coefficient of variation, and sensitivity for 2022 and 2023.

		Data number	Ppmax			Vstem		
			SI	CV	S	SI	CV	S
2022	New shoots	38	1.92a	0.31	6.16	0.59b	0.32	2.05
	Fruit expanding	78	2.05a	0.43	4.75	0.51a	0.43	1.20
	Fruit maturing	16	2.35a	0.50	4.73	0.64b	0.28	2.32
	Whole growth season	132	2.11a	0.41	5.20	0.58b	0.34	1.57
2023	New shoots	44	1.70a	0.22	7.71	1.26a	0.77	1.64
	Fruit expanding	76	1.96a	0.28	7.10	1.03a	0.49	2.09
	Fruit maturing	16	1.57a	0.35	4.44	0.93a	0.29	3.26
	Whole growth season	136	1.74a	0.28	6.15	1.07b	0.51	2.09

In the table, SI represents signal strength, CV represents coefficient of variation, S represents sensitivity, and the data shown are the mean values of each growth period. a and b indicate the difference between SI_Ppmax_ and SI_Vstem_(*p*<0.05).

### 3.4 Relationship between Ppmax and Vstem and meteorological factors

**Figure 3:**
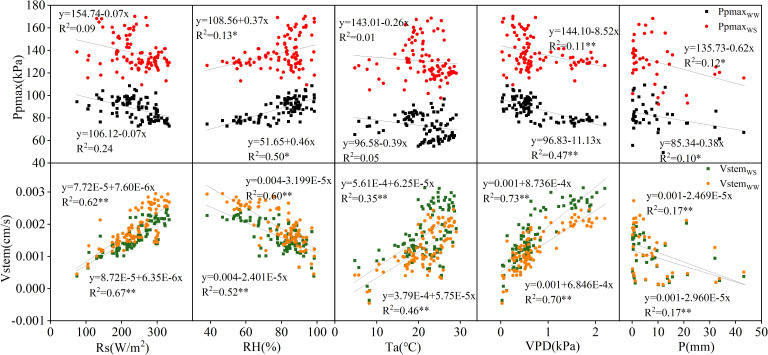
Correlation of Ppmax and Vstem with meteorological factors in 2022.

**Figure 4:**
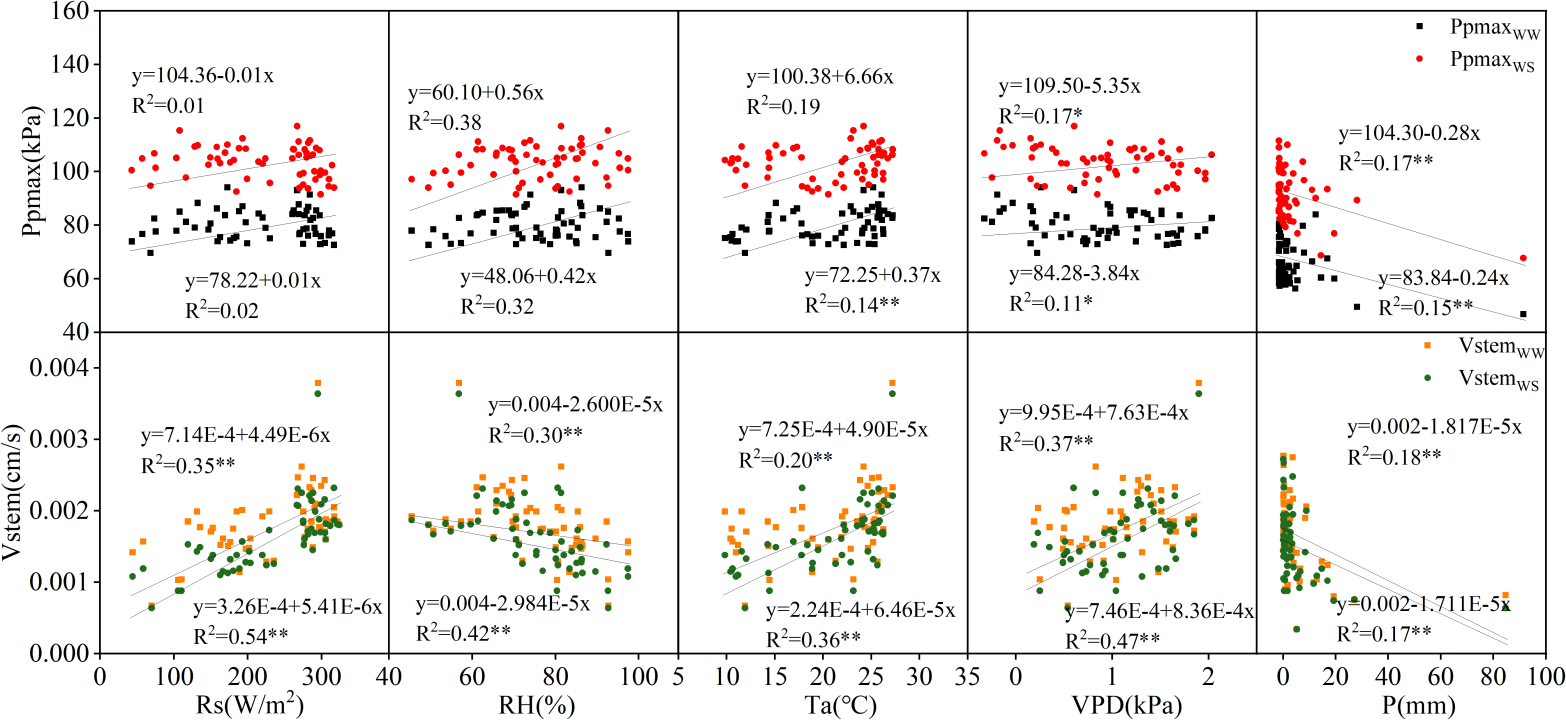
Correlation of Ppmax and Vstem with meteorological factors in 2023.

The correlations between Ppmax and Vstem and the meteorological factors in 2022 and 2023 were analyzed, and the results are shown in **Figures 3**, **4**. In 2022, Ppmax in the WW and WS treatments was negatively correlated with Rs, Ta, and VPD and positively correlated with RH. A negative correlation was observed between the Ppmax and rainfall. In general, Ppmax treated with WW had a high correlation with meteorological factors (0.05–0.50), and it had the highest correlation with RH, followed by VPD, and the lowest correlation with Ta. However, the response of the WS treatment to meteorological factors differed, relative humidity and rainfall were the main factors influencing Ppmax under inadequate irrigation. Compared with Ppmax, the determination coefficients of Vstem and various meteorological factors significantly increased and reached a very significant level (*p* < 0.01), which was unrelated to the irrigation treatment. The correlation between Vstem and VPD was the highest, indicating that VPD is the main meteorological factor causing changes in the stem flow rate in apple trees. In 2023, the Ppmax of the two treatments was positively correlated with RH and Ta and negatively correlated with VPD and P. Except for the correlation between Ppmax and RH in the WW treatment, the correlation coefficients between Ppmax and meteorological factors were lower than those between Vstem and meteorological factors, and this difference was more significant under inadequate irrigation. The WW-treated Vstem showed a strong correlation with Rs, followed by VPD, and a weak correlation with P. The effect of VPD on the Vstem of apple trees in the WS treatment was greater than that of Rs.

### 3.5 Relationship between Ppmax and Vstem and soil and leaf water potential

**Figure 5:**
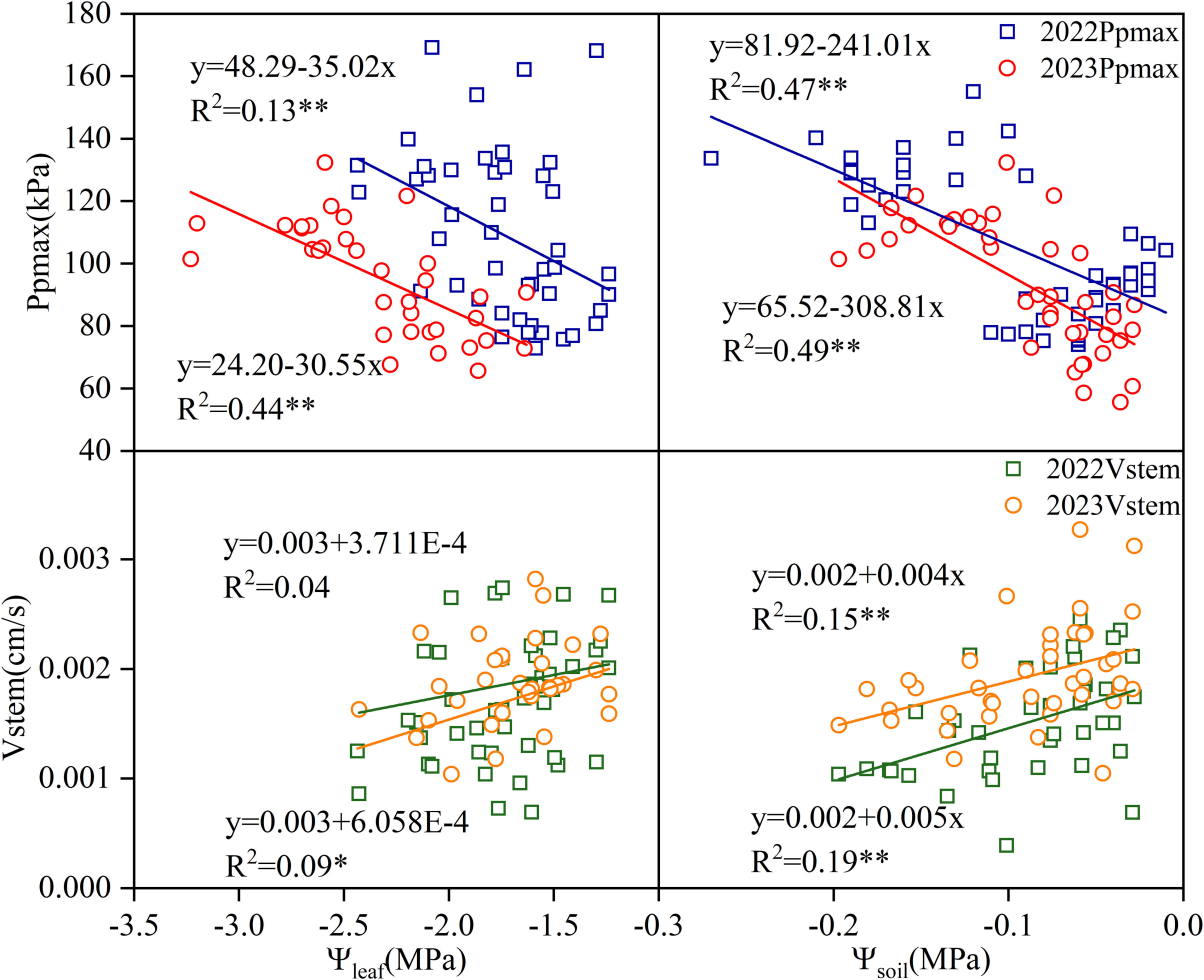
Correlation between two-year Ppmax, Vstem and leaf water potential and soil-water potential.

In both test years, Ppmax was significantly negatively correlated with leaf and soil water potential (*p* < 0.01), whereas Vstem was positively correlated with leaf and soil water potential (**Figure 5**). In 2022, the correlation between Vstem and Ψ_leaf_ was low and did not reach statistical significance (*p* > 0.05). There was a significant correlation between Vstem and Ψ_leaf_ in 2023, but its coefficient of determination was 4.89 times lower than those of Ppmax and Ψ_leaf._ The correlation between Vstem and Ψ_soil_ reached a significant level in both years (*p* < 0.01), but the correlation coefficient was significantly lower than that between Ppmax and Ψ_leaf_.

## 4 Discussion

### 4.1 Ppmax change patterns

Leaf turgor pressure plays a key role in plant water physiology. Water absorption and expansion of plant cells increase turgor pressure, whereas cell water loss causes protoplasts to contract, resulting in a decrease in turgor pressure (Crabos et al., 2023). In this study, the full irrigation treatment was applied in mid-May; therefore, the Ppmax values for the full irrigation treatment were significantly lower than those for the water deficit treatment on and after May 28, indicating that soil water deficit reduced leaf turgor pressure. Because the expansion of leaf cells mainly depends on water absorption and transportation in tree bodies, low soil water may limit the water absorption of roots, resulting in reduced hydraulic conductivity, leaf water loss, and turgor pressure (Thalheimer et al., 2024).

In the 2022 and 2023 trial years, the Ppmax of apple trees under adequate water conditions exhibited an initial increasing trend followed by a subsequent decrease throughout the entire growth period, primarily influenced by the intrinsic growth characteristics of the apple trees. Canopy coverage during the growing season is a major factor driving water utilization in apple orchards (Mupambi, 2017). Smaller leaf area resulted in a low water consumption intensity during the early stages of the experiment. As the growth period advanced, the leaf area of fruit trees reached the highest, and the enhances of transpiration demand and nutrient requirements led to increased leaf water consumption and Ppmax. At the same time, the beginning of fruit development increased the osmotic load of phloem, which affected the water storage dynamics of trees (Perez-Arcoiza et al., 2022). At the fruit maturity stage, the increase in abscisic acid content promoted stomatal closure, which reduced leaf transpiration and moisture loss while enhancing leaf turgor pressure. Therefore, Ppmax decreased and then returned to its initial level. Different from the change of leaf turgor pressure under full irrigation, Ppmax under water deficit treatment did not fully recover at the end of growing season. The reason may be that the low water content of the tree could not meet the demand of leaf turgor pressure recovery in the later stage of the experiment. A similar phenomenon can be observed in olive trees (Marino et al., 2021). However, Bader et al. (2014) found that the turgor pressure of barley leaves did not shift upward from the baseline, indicating that turgor pressure fully recovered at night, mainly because the tight stomatal regulation may help prevent excessive dehydration of leaves. In addition, the flow of phloem to fruit will be reduced or the xylem return will be increased when trees reach severe water deficit, which may also have an impact on leaf water (Tao et al., 2023).

The Ppmax of apple trees in 2022 fluctuated more sharply than that in 2023, due to the difference in leaf water content caused by different rainfall in the two years. Zimmermann et al. (2010) proved that maintaining good water conditions are critical for plant Pp oscillations. The results of this study show that frequent rainfall in 2022 could provide sufficient water for fruit trees. However, drought in 2023 limited the absorption and transport of water in the leaves and thus weakening the changes in leaf turgor pressure. This was stimulated by endogenous factors such as reduced root water conductance under drought stress, ABA accumulation, changes in guard cells, aquaporin-mediated hydraulic conduction, and transpiration potential across the root-hair boundary (Bramley et al., 2013). Previous studies have reported fluctuations in Pp signal in grape and banana plants, attributed to stomatal changes (Dzikiti et al., 2007; Westhoff et al., 2009). Due to physical or chemical reasons, stomata opening leads to increased water conductivity, water loss, and ultimately reduced leaf turgor pressure (Zimmermann et al., 2013). Furthermore, there was a strong correlation between the frequency (and amplitude) of the Pp oscillations and wind speed. The increase in wind speed significantly reduced the boundary layer on the leaves, which, in turn, temporarily increased local transpiration and thus reduced turgor pressure (Zimmermann et al., 2010).

### 4.2 Ppmax had superior signal strength and sensitivity

We investigated for the first time the difference in signal intensity, variability and sensitivity between Ppmax and Vstem to evaluate the reliability of Ppmax in indicating the water status of apple trees. In this study, Ppmax was found to be more suitable than Vstem as an indicator of water quality in apple trees. It was previously reported that Vstem is a reliable and widely used parameter for characterizing plant water status (Kamakura et al., 2021; Wu et al., 2024). However, in this experiment, SI_Ppmax_ was consistently higher than SI_Vstem_ throughout the apple tree growth stage in 2022 and 2023, with a maximum difference of 4.02 times. This may be related to the water transport capacity of leaves and stems. Some studies have found that plant leaves lose water transport efficiency earlier than that of stems during drought. This was due to the coordination between the reduction of hydraulic conductivity of the leaf vascular and extra-vascular level and the turgor pressure loss by leaf cells resulting in stomatal closure (Lo Gullo et al., 2003). However, the leaf turgor pressure can quickly recover when soil water availability is restored, suggesting that damage to leaf cells or water transport systems can be effectively reversed at the end of the dry period. This was mainly because the decrease in leaf hydraulic conductivity is not only the result of venous embolization but also cell contraction of the extracellular xylem pathway, and the consequent increased resistance may play an important role (Savi et al., 2016). Moreover, the fluctuation of SI_Ppmax_ during the entire growing season was between 0.39 and 0.43, which was much greater than the fluctuation of SI_Vstem_ in the growing season of apple trees. This indicated that Ppmax responds more sensitively to difference in soil moisture and saturated water pressure. This may be because the intensity of water storage and consumption in leaves was greater than that in stems, resulting in more frequent contraction and expansion of leaf guard cells observed at later stages of growth (Du et al., 2017). Based on the hydraulic segmentation hypothesis, leaves generally show higher vulnerability and are more prone to embolisms than stems. Therefore, trees may rely on leaf water consumption or shedding under drought conditions to maintain the stem hydraulic function (Bryant et al., 2021; Li et al., 2024). After embolization, repair is required to avoid the accumulation of nonfunctional catheters and complete loss of water conductivity, manifested by increased tissue water content (Bucci et al., 2012).

In 2022, Ppmax had more variability than Vstem, but the lower signal strength of Vstem led to lower sensitivity compared to Ppmax. The leaf turgor changes via back pressure of the common epidermal cells, for example, feedback on fine regulation of the stomatal aperture. On the one hand, frequent rainfall during the fruit ripening stage could cause the changes in atmospheric humidity and preferentially influence on leaf stomatal size, resulting in an increased Ppmax fluctuation frequency under inadequate irrigation conditions (Binstock et al., 2024). On the other hand, the absorption of large amounts of water by fruit trees caused changes in ion uptake, pH in the xylem sap, and chemical signals, which induced changes in stomatal aperture (Barragán et al., 2012). By 2023, the variability and sensitivity of Ppmax were better than those of Vstem, and this result has not been widely reported. It has been pointed out that stem flow occurs only when the xylem tension gradient exceeds a certain threshold; therefore, there will be a lag in the start or peak time of the stem flow rate (Rodriguez-Dominguez et al., 2012). Moreover, water in the stems of woody plants is mainly stored in the elastic bark tissue, indicating that the initiation of stem flow is limited by structural resistance, such as cell membranes and intercellular strands (Wan et al., 2023; Wang et al., 2023). Therefore, it is reasonable to assume that leaves and stems do not form a strictly coupled water flow system within a short time range, which may also be the reason for the rapid leaf turgor change and delayed stem flow feedback. In conclusion, based on the signal strength theory, Ppmax was a superior diagnostic indicator of water status in fruit trees compared to Vstem.

### 4.3 Ppmax was closely related to soil and fruit tree moisture

Changes in the moisture content of fruit trees closely related to meteorological conditions. Xu et al. (2024) concluded that on sunny days, the Pp of apple trees presented a diurnal variation curve with high values during the day and low values at night. In contrast, the variation amplitude decreased significantly, and the peak value decreased on rainy days. Therefore, different weather conditions cause differences in leaf turgor pressure. Based on the relationship between Ppmax and Vstem and meteorological factors under different weather conditions, we found that RH and VPD were the main factors affecting Ppmax in apple trees in 2022. In 2023, relative humidity was positively correlated with Ppmax, and VPD negatively affected Ppmax, which may be due to the isohydric behavior of apple trees. When the saturated water vapor pressure deficit increases, accompanied by a decrease in relative humidity, stomatal closure restricts water exchange with the outside world, resulting in a relatively constant leaf water potential. At this moment, the turgor pressure exhibited a steady or rising trend, leading to a decrease in Ppmax (Zhao et al., 2023). A similar phenomenon has been observed in pear trees by Kaneko et al. (2024). When water was insufficient, the isohydric behavior of pear leaves helped minimize the negative change in the water balance, thus reducing the adverse impact on the fruit. In contrast, the correlation between Vstem and meteorological factors increased significantly in both years, with minimum and maximum determination coefficients of 0.17 and 0.73, respectively. This indicated that the stem flow rate of apple trees is more sensitive to changes in meteorological conditions. In contrast, Ppmax was less affected by meteorological factors, and its changes were more attributable to the water supply and loss of fruit trees. These results are similar to those of Ehrenberger et al. (2012), who found that stem water deficit and Pp were significantly dependent on weather conditions in young oak trees; however, stem water deficit was significantly more sensitive to changes in VPD than Pp. This most likely reflects a hierarchical order of water partitioning within trees giving more preference to water demanding leaves than to water storing bark tissue to keep the leaf water content in an optimal range for physiological functions, such as photosynthetic carbon sequestration.

Additionally, the results of this study showed that Ppmax was significantly correlated with soil water potential and morning leaf water potential, supporting the view that Ppmax is a better indicator of apple tree water status than Vstem. Leaf turgor pressure is the pressure exerted on the cell wall by protoplasts within plant cells (Blackman, 2018). Turgor changes in guard cells are mediated by transport of solute (K^+^). Based on the water activity feedback hypothesis, the positive regulation of the osmotic pressure of guard cells in proportion to leaf turgor pressure may explain the close relationship between Ppmax and leaf water potential (Rodriguez-Dominguez et al., 2016). Some scholars have found in research on various tree species and crops that Pp gradually decreased with an increase in leaf water potential or noon stem water potential (Fernandez et al., 2011), which is similar to the results of our study. However, Vstem has a lower correlation with soil and leaf water potential because stem flow is not only affected by evapotranspiration and environmental conditions but is also limited by canopy coverage (Fu et al., 2022; Niessner et al., 2024). Thes conclusions are significant for the management of deficit irrigation strategies. Furthermore, turgor pressure is an internal factor that induces stomatal closure under water stress, which may further affect the photosynthetic capacity and yield of plants (Aaron and Xue, 2022; Miranda et al., 2022). It has been pointed out that different mesophyll cells will lose filling under different water conditions. In particular, stomatal protective cells are able to maintain higher turgor pressure than other epidermal cells, which may delay the complete closure of stomata under drought conditions (Franks and Farquhar, 2007). Therefore, the relationship between leaf turgor pressure and stomatal opening and closing should be further analyzed from the aspects of cell tissue and anatomical structure.

## 5 Conclusion

Monitoring the Ppmax of apple trees under different soil water conditions showed that the Ppmax of the full irrigation treatment was significantly lower than that of the under-irrigation treatment, and the average Ppmax of the water deficit treatment was 61.97% and 27.27% higher than that of the full water treatment. Ppmax treated with water deficiency did not recover completely at the end of the growing season. There were significant differences between SI_Ppmax_ and SI_Vstem_ in apple trees, as shown by SI_Ppmax_ was higher than SI_Vstem_ at each growth stage. In comparison to Vstem, Ppmax exhibited superior and sensitivity to variations in soil water content, demonstrating a lower correlation with meteorological factors but a stronger association with both soil moisture and fruit tree water content. In the future, it will be necessary to explore the relationship between Ppmax, the opening and closing of fruit tree stomata, and photosynthesis intensity further. This of great importance to explain the growth mechanisms of fruit trees comprehensively.

## Data Availability

The original contributions presented in the study are included in the article/supplementary material. Further inquiries can be directed to the corresponding author.
